# Occupational status as a determinant of mental health inequities in French young people: is fairness needed? Results of a cross-sectional multicentre observational survey

**DOI:** 10.1186/s12939-017-0634-7

**Published:** 2017-08-08

**Authors:** Marie Blanquet, Emilie Labbe-Lobertreau, Catherine Sass, Dominique Berger, Laurent Gerbaud

**Affiliations:** 1 Service de Santé Publique, Centre Hospitalier Universitaire de Clermont-Ferrand, 7, place Henri Dunant CEDEX 1, 63058 Clermont-Ferrand, France; 2 Clermont Université, Université d’Auvergne, EA 4681, PEPRADE (Périnatalité, grossesse, Environnement, PRAtiques médicales et DEveloppement), Clermont-Ferrand, France; 3 Centre Technique d’Appui et de Formation des Centres d’Examens de Santé, Cetaf, 67/69 Avenue de Rochetaillée, 42100 Saint-Etienne, France; 4 Université Claude Bernard Lyon 1 – ESPE, Université de Lyon, HESPER – Health Services and Performance Research, 5 rue Anselme, Cedex, 69004 Lyon, France

**Keywords:** Health inequities, Occupational status, Young people, Self-perceived health, Mental health

## Abstract

**Background:**

Employment conditions are associated with health inequities. In 2013, French young people had the highest unemployment rate and among those who worked as salaried workers most of them had temporary job. The purpose of the study was to assess mental health state of French young people through the prism of their occupational status and to measure whether occupational status is a determinant of health inequities.

**Methods:**

A cross-sectional multicentre observational survey was performed in June and July 2010 in 115 French Local Social Centres and 74 Health Examination Centres, who were available to participate. The survey was based on an anonymous self-administrated questionnaire delivered by social workers or healthcare professionals to young people age from 16 to 25 years old. The questionnaire was composed of 54 items. Several health outcomes were measured: self-perceived health, mental health, addictions and to be victim of violence. The association of occupational status and mental health was assessed by adjusting results on age and gender and by introducing other explanatory variables such as social deprivation.

**Results:**

A total of 4282 young people completed the questionnaire, a response rate of 83%, 1866 men and 2378 women, sex-ratio 0.79. French young people having a non-working occupational status or a non-permanent working status were more exposed to poor self-perceived health, poor mental health, addictions and violence. To be at school particularly secondary school was a protective factor for addiction.

**Conclusions:**

Occupational status of French young people was a determinant of mental health inequities. Young people not at work and not studying reported greater vulnerability and should be targeted therefore by appropriate and specific social and medical services.

**Electronic supplementary material:**

The online version of this article (doi:10.1186/s12939-017-0634-7) contains supplementary material, which is available to authorized users.

## Background

The World Health Organization-Europe (WHO) has defined social inequities in health as “systematic differences in health status between socioeconomic groups, as measured by income, education and occupation that are, within a country, socially produced, modifiable and unfair” [1]. As there are determinants of health defined by factors that influence health positively or negatively, there are also determinants related to social, economic and life-style conditions that play a role in health status. They are conceptualized by macro and micro-conceptual frameworks [1, 2]. The micro-conceptual framework depicted influence employment conditions on health inequities through working conditions, material deprivation and economic inequities and psychosocial factors [3]. The relationship between employment, working conditions, and adverse health outcomes, low self-perceived health [4–8], bad and poor physical health [8–11], and mental health [9–13], have been previously explored by several studies.

The concept of deprivation was both defined by Wrezinski as “the lack of safety, like a job, enabling individuals and families to assume professional, family and social responsibilities and to enjoy basic rights” and by Townsend as a “state of observable and demonstrable disadvantage relative to the local community or the wider society to which an individual, family or group belongs” and identified as the main cause of inequities in health [14, 15]. As a continuum, precarious employment might be defined as “a multidimensional construct encompassing dimensions such as employment insecurity, individualized bargaining relations between workers and employers, low wages and economic deprivation, limited workplace rights and social protection, and powerlessness to exercise workplace rights” [16]. Even if no consensus exists on its definition, precarious employment appears to be an emerging social determinant of health that Benach et al. put at a central place in their conceptual model with straight influence on hazardous working conditions, material deprivation, and health and quality of life. This central place warrants the needs of further research [16].

The most recent French study on labour market made by the National Institute of Statistics and Economic studies (Insee), in accordance with the International Labour Organization (ILO) definitions, was performed in 2013. This survey, based on data recorded in 2013, showed highest unemployment rate in young people aged 15–24 years old, 23.9% vs. 9.1% in the 25–49 year old group and 6.5% in people aged 50 years old or over with an annual average unemployment rate of 9.8% [17]. Young people had also the highest underemployment rate 11.7% vs. 6.2% in the 25–49 year old group and 5.7% in person aged 50 years old or over [17]. They worked as salaried workers more frequently than the economically active population (97.5% vs. 88.8%) with greater proportion of young people working in interim (5.9% vs. 2.0%), in training school (18.1% vs. 1.6%) and in temporary job (28.3% vs. 8.4%) [17].

Taking into consideration this context, the National Council of Local Social Centres (Conseil National des Missions Locales- CNML), who has the mission to understand and improve integration of young people into the work place mechanisms, asked the Support and Education Technical Centre of Health Examination Centres (Centre technique d’appui et de formation des centres d’examens de santé-CETAF), for a survey on young people’s health through their occupational status.

The main aim of our survey was to assess mental health state of French young people through the prism of their occupational status. The secondary aim was to measure whether occupational status of French young people is a determinant of health inequities. Specifically, self-perceived health, mental health, addictions and violence were assessed by adjusting results on sociodemographic variables and by considering childhood major event, social support, and health care consumption.

## Methods

### Setting

In France, 447 Local Social Centres (LSC), created in 1982 (Order n°82–273, March 1982), provide social, health and education support to young people without qualification, to be integrated into the workplace. Since its creation, 1,400,000 of young people were taken in each year. There also are 112 preventive Health Examination Centres (HEC), created in 1945 (Article L321–3 Health insurance Code). They provide preventive medical consultations to beneficiaries of the national health insurance for salaried workers and their family. Patients do not pay and can consult without being referred. In 1992, a ministerial order designated who should have priority for these consultations, such as retirees, job seekers, young people just entering the job market, the homeless and people exposed to risk factors for health [18]. These people account for a third of all those annually cared for by the HECs.

### Methods

A cross-sectional multicentre observational survey was performed in June and July 2010 in 115 French LSC and 74 HEC. LSC and HEC were free to participate.

The survey was based on an anonymous self-administrated questionnaire delivered by social workers or healthcare professionals working in the LSC or HEC. Professionals explained to young people the aim of the survey and were available to answer questions young people could have. The survey was carried out during 2 weeks per LSC or HEC.

The questionnaire was elaborated by a working group between 2007 and 2008. The working group was composed by the CNML, the French national health insurance for salaried workers (Caisse Nationale d’Assurance Maladie-travailleurs salariés- CNAMts), Health Directorate (Direction Générale de la Santé-DGS), Social Welfare Directorate (Direction Générale de l’Action Sociale- DGAS), the CETAF and representatives of LSC and HEC. A first version of 68 items has been elaborated based on questionnaires previously used in the European School Survey Project on Alcohol and Other Drugs (ESPAD), the French Health Barometer and the survey of the French Monitoring Centre for Drugs and Drug Addiction (Observatoire Français des drogues et des Toxicomanies-OFDT). The first version was tested in 24 LCS and 14 HEC on 1342 questionnaires. The second version was then elaborated and used in the present work.

### Population

People included were young people age from 16 to 25, coming from LSC or HEC. There were no exclusion criteria. The first 15 to 30 people who were taken in charge by social workers in LSC and health care professionals in HEC were asked to participate in the study. All participants gave their informed consent to be enrolled.

### Questionnaire

The questionnaire was composed by 54 items on socio-demographic status, deprivation, addictions, psychological disorders, health state, violent behaviour and discrimination and the need of medical, social or family support. The socio-demographic status included 21 items: age, sex, home location, own and parent’s current occupational status, level of education, social support, medical insurance, and serious childhood life events. Details on young people’ occupational status, are displayed in the Additional file 1. Deprivation was measured by the EPICES score, a validated individual score of individual social deprivation [19, 20]. Addictions included items about alcohol; consumption in the last 30 days and drunkenness in the last 12 months, current tobacco consumption and marijuana consumption in the last 30 days and feeling of need to smoke marijuana to feel health since the morning. Life events during childhood were measured by 4 items: have lived family events as break-up, disease, depression, alcoholism etc.; have had accident or serious illness; have been involved in an educative or judicial intervention; how was the childhood and adolescence. Psychological disorders were measured by two validated questionnaires, the Mental Health Index (MHI-5) and the Adolescent Depression Rating Scale (ADRS), and by items about suicide [21]. Health state was also measured by the scale of self-perceived health. Three kinds of violence behaviour, psychological, physical and sexual, as victim or perpetrator were assessed. Discrimination in different place (employment, accommodation, leisure, education and transports) was also integrated into the questionnaire. Then, people were questioned about needs for medical, social or family support they had had during the past 12 months (Additional file 2).

### Health outcomes

Nine health outcomes were measured: self-perceived health, mental health, addiction and to be victim of violence. Self-perceived health was a validated and relevant measurement of health predicting mortality [22, 23]. It was measured through a numeric scale from 0 for the poorest self-perceived health to 10 for the best self-perceived health. The threshold of 7 was used with poor self-perceived health under 7 [24, 25]. Mental health was assessed by the Mental Health Inventory- 5, a dimension of the well-known and validated SF-36, with psychological distress under 56 and a question about suicide attempt. Addiction to three substances was measured: current tobacco use, alcohol misuse (when young people reported being drunk at least three times in the last year) and marijuana use. Exposition to three kind of violence was explored, psychological, physical and sexual violence.

### Explanatory variables

The main explanatory variable assessed, as in occupational status of French young people, was composed by 13 categories: permanent job, temporary job, interim, specific employment contract, to be at school or secondary school, to study at university or out of university, training school, to be in block release training school, trainee in education, in integrating into the work place, job seeking or unemployed. The other explanatory variables were level of education (low when <2nd school diploma and high when >2nd school diploma), major event in childhood (meaning yes if the person reported at least one kind of major event during his childhood), deprivation (through the calculation of EPICES score), discrimination (i.e.-if the person declared being victim of at least one kind of discrimination), financial social support (i.e.-if the person received at least one kind of financial support), social worker support (if the person met a social worker during the last 12 months), social network support (if the person met family, friends or other relations in the last 12 months), and health care consumption (meaning the person met a health care professional in the last 12 months).

### Statistical analysis

Descriptive analysis was performed to assess the sample characteristics by using percentage for qualitative variables and means with standard deviation for quantitative variables. Multivariate analysis was performed by using stepwise forward logistic regression adjusted on age and gender to assess health outcomes through three steps. The first step integrated the occupational status as explanatory variables adjusted on age and gender. The second step added all the other explanatory variables, excepting individual social deprivation measured by the EPICES score. The third step added all the explanatory variables statistically significant at the second step and the EPICES score, to identify information this explanatory variable specifically brought into the model. Logistic regression models identified meaningful explanatory variables based on the calculation of the adjusted Odds Ratio (OR) and its 95% Confidence Interval [95%CI]. The R^2^ of Nagelkerke describing the appropriateness of fit of the model was presented. A meaningful threshold of 5% was chosen for all the statistical analyses. Statistical analysis was performed on SPSS V 16.0 software.

## Results

A total of 4282 young people filled in the questionnaire, 1866 men and 2378 women, sex-ratio 0.79 and 8% refused to participate in (Table 1).

**Table 1:** Descriptive analysis in the whole sample and comparisons by gender

	%^a^ m^b^ [sd]^c^ *(n* ^*d*^ *)* Total; *N* = 4282	%^a^ m^b^ [sd]^c^ *(n* ^*d*^ *)* Men; *N* = 1866	%^a^ m^b^ [sd]^c^ *(n* ^*d*^ *)* Women; *N* = 2378	*p*-value
Age	20.91 [2.59] *(4235)*	20.75 [2.63] *(1860)*	21.03 [2.54] *(2375)*	<0.001
Level of education (low)^e^	68.6 *(4188)*	74.4 *(1822)*	64.0 *(2329)*	<0.001
Occupational status	*(4212)*	*(1850)*	*(2360)*	<0.001
Permanent job	6.0	5.7	6.2	
Temporary job	*4.7*	*3.8*	*5.3*	
Interim	*3.4*	*5.4*	*1.9*	
Specific employment contract	2.7	*2.1*	*3.2*	
School/secondary school	8.8	8.5	9.0	
At university	3.5	2.9	4.0	
Out of university	3.0	1.5	4.2	
Training school	2.5	4.1	1.4	
Block release training school	4.0	4.5	3.5	
Trainee in education	10.9	10.5	11.2	
In integrating into the work place	5.7	7.1	4.5	
Job seeking	36.3	35.4	37.1	
Unemployed	8.5	8.6	8.5	
Major event in childhood (yes)	61.5 *(4253)*	62.5 *(1849)*	60.6 *(2368)*	*ns* ^*h*^
EPICES score quantitative	40.17 [20.34] *(3791)*	40.29 [19.46] *(1627)*	40.08 [21.04] *(2133)*	*ns* ^*h*^
EPICES score qualitative^i^	65.7 *(3791)*	*66.7 (1627)*	*65.0 (2133)*	*ns* ^*h*^
Discrimination (yes)	25.1 *(4120)*	25.1 *(1782)*	25.0 *(2314)*	*ns* ^*h*^
Financial Social support (yes)	79.0 *(3379)*	78.7 *(1863)*	79.2 *(2377)*	*ns* ^*h*^
Social worker support (yes)	17.4 *(3991)*	16.6 *(1720)*	18.0 *(2247)*	*ns* ^*h*^
Social network support (yes)	38.5 *(4010)*	29.3 *(1723)*	45.4 *(2264)*	<0.001
Family physician (yes)	77.2 *(4248)*	69.1 *(1847)*	83.5 *(2364)*	<0.001
Health care consumption (yes)	33.9 *(4072)*	26.6 *(1755)*	39.3 *(2293)*	<0.001
Health outcomes				
Self-perceived health (<7)	26.7 (4214)	23.2 (1835)	29.6 (2342)	<0.001
MHI-5^f^ (< 56)	38.2 (4133)	29.8 (1786)	44.9 (2310)	<0.001
Suicide attempt (yes)	14.7 (4129)	10.1 (1783)	18.2 (2325)	<0.001
Tobacco smoke (yes)	50.6 (4243)	58.4 (1847)	44.4 (2360)	<0.001
Alcohol misuse^g^	20.6 (4049)	29.8 (1768)	13.4 (2246)	<0.001
Marijuana use	20.4 (4206)	29.4 (1833)	13.4 (2338)	<0.001
Victim of psychological violence (yes)	34.2 (4062)	29.8 (1760)	37.5 (2281)	<0.001
Victim of physical violence (yes)	29.6 (4036)	31.4 (1760)	28.1 (2253)	0.03
Victim of sexual violence (yes)	10.1 (3988)	3.4 (1720)	15.2 (2247)	<0.001

^a^ *percentage;* ^*b*^ *mean;* ^*c*^ *standard deviation;* ^*d*^ *number of person with a response to the question;* ^*e*^ *less than 2nd school diploma;* ^*f*^ *Mental Health Inventory;* ^*g*^ *≥ 3 drunkenness in the last year;* ^*h*^ *ns: non statistically significant; i: EPICES score > 30.17 = social deprivation*

In the main four occupational statuses the young people included had, three were non-working statues, with in decreasing order: job seeker (36.3%), trainee in work education (10.9%), and to be unemployed (8.5%). Occupational statuses were significantly different in men and women (*p* < 0.001) with higher percentage of men working in interim (5.4% vs. 1.9%), being in training school (4.1% vs. 1.4%), and undergoing integration into the work place (7.1% vs. 4.5%) and lower percentage of men studying out of university (1.5% vs. 4.2%). A total of 61.5% of the sample reported childhood life event. EPICES score mean was at 40.17 (standard deviation 20.34) and 79.0% declared having financial social support. A third of the sample (33.9%) reported health care consumption with significant different behaviour by gender (men 26.6% vs women 39.3%; *p* < 0.001) (Table 1).

Concerning global perception of health, 26.7% of young people included reported a poor self-perceived health, women in particular (men 23.2% vs. women 29.6; *p* < 0.001). Women experienced poor low mental health with higher percentage of young women in psychological distress (men 29.8% vs. women 44.9%, *p* < 0.001) and with medical history of suicide attempt (men 10.1% vs. women 18.2, *p* < 0.001). Concerning addiction, half of the sample were current smokers (50.6%), 20.6% reported alcohol misuse with at least three drunkenness in the last year, and marijuana consumption with statistically significant difference by gender. Women reported higher exposition to psychological and sexual violence than men. On the contrary, men declared more physical violence than women (Table 1).

In the first step of the multivariate analysis, young people being unemployed, job seekers, undergoing integration into the work place, trainee in education and in block release training school, reported more adverse health outcomes than those working. They declared higher level of poor self-perceived health, more psychological distress, more suicide attempts, more tobacco and marijuana use and more violence irrespective of the type. In young people who worked, those in specific employment contract, declared poor self-perceived health more often, suicide attempts, tobacco use and reported being the victim of psychological violence more often than those who had permanent job. Young people at school or at secondary school were less addicted to tobacco, alcohol and marijuana than those who had permanent job (Tables 2 and 3).

**Table 2:** Multivariate analysis including the occupational status as sole explanatory variable adjusted on age and gender

Explanatory variables		Self-perceived health <7 *N* = 4144 OR^a^ [95%CI^b^]	MHI-5 < 56 *N* = 4063 OR [95%CI]	Suicide attempt *N* = 4073 OR [95%CI]	Tobacco smoke *N* = 4167 OR [95%CI]	Alcohol misuse *N* = 3982 OR [95%CI]	Marijuana use *N* = 4134 OR [95%CI]
Age		**1.05 [1.02; 1.09]**	**1.04 [1.01; 1.08]**	0.98 [0.93; 1.02]	0.98 [0.95; 1.01]	0.98 [0.94; 1.01]	0.97 [0.94; 1.01]
Gender	Men	1	1	1	1	1	1
	Women	**1.44 [1.24; 1.66]**	**1.97 [1.72; 2.26]**	**2.06 [1.70; 2.50]**	**0.59 [0.51; 0.67]**	**0.37 [0.31; 0.44]**	**0.38 [0.32; 0.45]**
Occupational status	Permanent job	1	1	1	1	1	1
	Temporary job	1.19 [0.73; 1.93]	1.30 [0.86; 1.95]	**2.20 [1.20; 3.40]**	1.30 [0.89; 1.91]	0.81 [0.50; 1.33]	1.33 [0.80; 2.20]
	Interim	1.66 [0.98; 2.78]	1.45 [0.91; 2.29]	1.59 [0.77; 3.24]	**1.68 [1.10; 2.58]**	0.77 [0.46; 1.28]	1.16 [0.68; 1.98]
	Specific employment contract	**1.94 [1.14; 3.29]**	1.48 [0.92; 2.37]	**2.05 [1.03; 4.08]**	**1.79 [1.13; 2.81]**	1.15 [0.66; 1.99]	1.57 [0.88; 2.79]
	School/secondary school	1.08 [0.67; 1.74]	0.98 [0.65; 1.47]	1.16 [0.62; 2.14]	**0.51 [0.34; 0.74]**	**0.50 [0.30; 0.81]**	**0.55 [0.32; 0.92]**
	At university	1.54 [0.93; 2.56]	1.40 [0.89; 2.18]	0.62 [0.26; 1.46]	**0.50 [0.31; 0.78]**	0.81 [0.47; 1.39]	0.78 [0.42; 1.42]
	Out of university	1.33 [0.77; 2.29]	1.08 [0.67; 1.74]	0.65 [0.27; 1.53]	0.76 [0.47; 1.20]	1.08 [0.62; 1.86]	1.15 [0.62; 2.10]
	Training school	1.67 [0.92; 3.02]	1.34 [0.79; 2.27]	1.47 [0.66; 3.27]	**1.96 [1.20; 3.21]**	1.24 [0.72; 1.13]	1.75 [0.99; 3.07]
	Block release training school	**2.46 [1.52; 3.96]**	**1.59 [1.02; 2.46]**	**2.13 [1.12; 4.04]**	**1.53 [1.01; 2.31]**	0.97 [0.58; 1.59]	1.53 [0.92; 2.55]
	Trainee in education	**2.09 [1.41; 3.11]**	1.26 [0.89; 1.79]	**2.16 [1.26; 3.68]**	**1.61 [1.16; 2.23]**	0.84 [0.56; 1.26]	**1.53 [1.009; 2.33]**
	In integrating into the work place	**3.42 [2.22; 5.26]**	**2.21 [1.49; 3.28]**	**2.68 [1.50; 4.78]**	**1.65 [1.13; 2.40]**	1.11 [0.71; 1.73]	**1.91 [1.20; 3.03]**
	Job seeking	**2.27 [1.59; 3.23]**	**1.85 [1.36; 2.50]**	**2.04 [1.25; 3.33]**	**1.54 [1.16; 2.05]**	**0.69 [0.48; 0.97]**	1.18 [0.81; 1.73]
	Unemployed	**2.26 [1.50; 3.40]**	**2.22 [1.55; 3.18]**	**3.08 [1.80; 5.28]**	**1.76 [1.25; 2.47]**	1.02 [0.67; 1.55]	**1.58 [1.02; 2.44]**

^a^ *OR: Odds Ratio;* ^*b*^ *95%CI: 95% Confident Interval*

Significant results are in bold

**Table 3:** Multivariate analysis including the occupational status as sole explanatory variable adjusted on age and gender

Explanatory variables		Victim of psychological violence *N* = 4006; OR^a^ [95%CI^b^]	Victim of physical violence *N* = 3977; OR [95%CI]	Victim of sexual violence *N* = 3935; OR [95%CI]
Age		**1.07 [1.03; 1.10]**	**1.03 [1.001; 1.07]**	**1.08 [1.03; 1.13]**
Gender	Men	1	1	1
	Women	**1.44 [1.25; 1.65]**	0.88 [0.76; 1.02]	**5.27 [3.94; 7.04]**
Occupational status	Permanent job	1	1	1
	Temporary job	1.39 [0.91; 2.11]	1.08 [0.69; 1.69]	0.94 [0.44; 1.99]
	Interim	**1.82 [1.15; 2.87]**	1.52 [0.95; 2.44]	1.70 [0.74; 3.89]
	Specific employment contract	**1.84 [1.13; 2.96]**	1.40 [0.84; 2.31]	1.33 [0.59; 2.95]
	School/secondary school	1.39 [0.92; 2.10]	1.15 [0.75; 1.76]	1.56 [0.78; 3.11]
	At university	1.30 [0.82; 2.07]	0.70 [0.41; 1.20]	1.17 [0.52; 2.60]
	Out of university	1.12 [0.68; 1.85]	0.77 [0.44; 1.35]	0.62 [0.23; 1.62]
	Training school	1.25 [0.72; 2.17]	1.07 [0.61; 1.88]	1.10 [0.34; 3.45]
	Block release training school	**1.79 [1.14; 2.80]**	**1.84 [1.16; 2.89]**	1.18 [0.52; 2.69]
	Trainee in education	**1.94 [1.36; 2.77]**	**1.59 [1.10; 2.30]**	**1.88 [1.04; 3.39]**
	In integrating into the work place	**2.56 [1.72; 3.81]**	**2.00 [1.32; 3.00]**	**2.83 [1.48; 5.41]**
	Job seeking	**1.71 [1.25; 2.33]**	**1.54 [1.12; 2.13]**	**1.87 [1.10; 3.16]**
	Unemployed	**1.88 [1.30; 2.73]**	**1.69 [1.15; 2.47]**	**3.13 [1.74; 5.61]**

^a^ *OR: Odds Ratio;* ^*b*^ *95%CI: 95% Confident Interval*

Significant results are in bold

In the last step of the multivariate analysis, occupational status remained as an explanatory variable for self-perceived health (R^2^ = 0.16) and addictions (R^2^ from 0.10 to 0.13). Poor self-perceived health was reported by young people in block release training school (OR = 1.76; 95%CI [1.01; 3.06]), and undergoing integration into the work place (OR = 1.99; 95%CI [1.21; 3.24]) more often. Young people in training school were more exposed to tobacco (OR = 2.25; 95%CI [1.29; 3.93]) and marijuana use (OR = 2.12; 95%CI [1.15; 3.89]). Young people at school and secondary school remained less exposed to addiction (tobacco smoke: OR = 0.53; 95%CI [0.34; 0.81], and alcohol misuse OR = 0.57; 95%CI [0.34; 0.95]). The main explanatory variables frequently associated to the other health outcomes were gender (female),major event in childhood, discrimination, being deprived, and higher needs of social worker support, social network support and health care consumption (Tables 4 and 5).

**Table 4:** Multivariate analysis including all explanatory variables for self-perceived health and addictions adjusted on age and gender

Explanatory variables		Self-perceived health <7 *N* = 3500; R^2a^ = 0.16 OR^b^ [95%CI]^c^	Tobacco smoke *N* = 3480; R^2^ = 0.13 OR [95%CI]	Alcohol misuse *N* = 3723; R^2^ = 0.10 OR [95%CI]	Marijuana use *N* = 3498; R^2^ = 0.13 OR [95%CI]
Age		1.03 [0.99; 1.07]	1.02 [0.98; 1.06]	0.93 [0.92; 1.01]	0.98 [0.94; 1.03]
Gender	Men	1	1	1	1
	Women	**1.36 [1.14; 1.61]**	**0.59 [0.50; 0.68]**	**0.34 [0.28; 0.40]**	**0.34 [0.28; 0.41]**
Occupational status	Permanent job	1	1	1	1
	Temporary job	0.81 [0.46; 1.41]	1.18 [0.77; 1.79]	0.80 [0.48; 1.32]	1.13 [0.65; 1.96]
	Interim	1.01 [0.55; 1.85]	1.31 [0.82; 2.10]	0.74 [0.43; 1.28]	0.95 [0.53; 1.69]
	Specific employment contract	1.07 [0.57; 1.96]	1.36 [0.82; 2.24]	1.08 [0.61; 1.90]	1.25 [0.66; 2.33]
	School/secondary school	0.90 [0.52; 1.55]	**0.53 [0.34; 0.81]**	**0.57 [0.34; 0.95]**	0.64 [0.36; 1.12]
	At university	1.59 [0.91; 2.79]	**0.59 [0.36; 0.97]**	0.59 [0.33; 1.05]	0.68 [0.35; 1.32]
	Out of university	1.42 [0.78; 2.58]	0.96 [0.59; 1.58]	0.90 [0.51; 1.59]	1.23 [0.65; 2.34]
	Training school	1.67 [0.86; 3.25]	**2.25 [1.29; 3.93]**	1.44 [0.82; 2.53]	**2.12 [1.15; 3.89]**
	Block release training school	**1.76 [1.01; 3.06]**	1.27 [0.80; 2.01]	1.11 [0.66; 1.87]	1.68 [0.96; 2.91]
	Trainee in education	1.29 [0.82; 2.03]	1.31 [0.91; 1.88]	0.89 [0.58; 1.35]	1.33 [0.84; 2.11]
	In integrating into the work place	**1.99 [1.21; 3.24]**	1.47 [0.97; 2.24]	1.24 [0.78; 1.96]	1.54 [0.93; 2.55]
	Job seeking	1.45 [0.97; 2.17]	1.30 [0.95; 1.78]	0.72 [0.50; 1.03]	1.00 [0.66; 1.50]
	Unemployed	1.21 [0.75; 1.94]	1.36 [0.92; 2.00]	1.11 [0.72; 1.72]	1.37 [0.84; 2.21]
Level of education	> 2nd school diploma		1	1	
	< 2nd school diploma		**1.53 [1.28; 1.82]**	**0.63 [0.51; 0.78]**	
Major event in childhood	No	1	1	1	1
	Yes	**1.31 [1.10; 1.57]**	**1.81 [1.55; 2.10]**	**1.42 [1.18; 1.70]**	**1.87 [1.53; 2.27]**
Social network support	No		1	1	1
	Yes		**1.36 [1.16; 1.58]**	**1.55 [1.30; 1.85]**	**1.59 [1.32; 1.90]**
Discrimination	No	1	1		
	Yes	**1.46 [1.22; 1.75]**	**0.71 [0.60; 0.85]**		
EPICES score		**1.02 [1.01; 1.03]**	**1.01 [1.005; 1.02]**		**1.01 [1.005; 1.02]**
Social worker support	No				
	Yes	**1.33 [1.07; 1.66]**			
Health care consumption	No	1			
	Yes	**1.89 [1.57; 2.26]**			

^a^ *R* ^*2*^ *: R* ^*2*^ *de Nagelkerke* ^*b*^ *OR: Odds Ratio;* ^*c*^ *95%CI: 95% Confident Interval*

Significant results are in bold

**Table 5:** Multivariate analysis including all explanatory variables for mental health and violence adjusted on age and gender

Explanatory variables		MHI-5 < 56 *N* = 3496; R^2*a*^ = 0.26 OR^*b*^ [95%CI] ^*c*^	Suicide attempt *N* = 3451; R^2^ = 0.22 OR [95%CI]	Victim of psychological violence *N* = 3473; R^2^ = 0.25 OR^a^ [95%CI^b^]	Victim of physical violence *N* = 3405; R^2^ = 0.19 OR [95%CI]	Victim of sexual violence *N* = 3424; R^2^ = 0.20 OR [95%CI]
Age		1.02 [0.98; 1.05]	0.97 [0.92; 1.01]	**1.04 [1.007; 1.08]**	1.02 [0.98; 1.06]	1.04 [0.98; 1.09]
Sex	Men	1	1	1	1	1
	Women	**1.81 [1.54; 2.12]**	**1.89 [1.51; 2.37]**	**1.19 [1.01; 1.40]**	**0.76 [0.64; 0.90]**	**4.75 [3.44; 6.56]**
Level of education	> 2nd school diploma		1		1	
	< 2nd school diploma		**1.46 [1.12; 1.88]**		**1.37 [1.13; 1.67]**	
Major event in childhood	No	1	1	1	1	1
	Yes	**1.34 [1.13; 1.58]**	**1.82 [1.42; 2.34]**	**1.89 [1.58; 2.34]**	**1.89 [1.57; 2.26]**	**2.23 [1.64; 3.02]**
Social network support	No	1	1	1	1	1
	Yes	**2.11 [1.79; 2.49]**	**1.79 [1.43; 2.24]**	**3.13 [2.64; 3.70]**	**2.05 [1.72; 2.45]**	**1.61 [1.24; 2.09]**
Discrimination	No	1	1	1	1	1
	Yes	**1.72 [1.44; 2.06]**	**1.36 [1.08; 1.70]**	**1.66 [1.38; 1.98]**	**1.53 [1.28; 1.84]**	**1.48 [1.14; 1.92]**
EPICES score		**1.03 [1.02; 1.04]**	**1.02 [1.01; 1.03]**	**1.02 [1.01; 1.03]**	**1.02 [1.01; 1.03]**	**1.02 [1.01; 1.03]**
Social worker support	No			1	**1**	**1**
	Yes		**1.40 [1.08; 1.80]**	**1.29 [1.03; 1.61]**	**1.53 [1.23; 1.91]**	**1.46 [1.08; 1.97]**
Health care consumption	No	1	1	1	**1**	**1**
	Yes	**2.08 [1.75; 2.46]**	**2.33 [1.85; 2.92]**	**1.59 [1.33; 1.90]**	**1.39 [1.15; 1.68]**	**1.56 [1.18; 2.04]**

^a^ *R* ^*2*^ *: R* ^*2*^ *de Nagelkerke* ^*b*^ *OR: Odds Ratio;* ^*c*^ *95%CI: 95% Confident Interval*

Significant results are in bold

## Discussion

### Main results

Occupational status of French young people was a determinant of poorer self-perceived health, addictions and mental health. Young French people who were either not at work, at work without a permanent status or studying in training school or block release training school, were more exposed to poor mental health outcomes.

It is also interesting to point out that to add the measurement of individual social deprivation through the EPICES score into the multivariate analysis undermined occupational status and especially the non-working categories. Non-working status categories therefore seemed measuring individual social deprivation too. On the other hand, greater odds ratio was observed for the category “training school” when EPICES score was added, for consumption of tobacco and marijuana.

Concerning addiction, the protective association of school, secondary school and university, was another point of interest. Indeed, to study at university was not protective for alcohol consumption revealing the impact of binge drinking. Non-working occupational status was not associated with higher alcohol misuse but on the contrary to lower alcohol misuse, job seeking in particular, which means that alcohol misuse seemed to be more significant into social groups.

### Comparisons with other studies

Non-working status categories were identified as a variable measuring individual social deprivation. This result was support by Benach et al. who explained in their work: “precarious employment is clearly related to absolute and relative social deprivation” [16].

Virtanen et al. revealed that unemployed included three categories; compensation-income, subsidy or low-income, expressed systematically poor self-rate health irrespective of the gender, which mirrors our findings [26]. This conclusion was also drawn for atypical employees defining by all form of work excepted permanent employees and fixed-term employees, as in our survey for young people being in interim or with a specific employment contract. Virtanen et al. deepened their results in a review, revealing an association between temporary employment and increased psychological morbidity [9]. László et al. showed an association between job insecurity and health in European countries, even though it was not significant in France [7]. Bambra et al. provided further evidence of such an association in the 2010 European Working Conditions Survey [8]. Employees having a temporary contract reported significant poor self-perceived health (OR = 1.24; 95%CI [1.13–1.37]). Two recent Italian studies also identified that people in unstable working conditions declared poorer self-perceived health than permanent workers [6, 27]. One of the studies revealed that this association existed among young people in particular [27]. Three previous French studies showed association between employment characteristics, full time vs. part time and permanent job vs. non-permanent job and adverse health outcomes like self-perceived health [28–30]. When comparing genders, studies identified that both men and women expressed more damage on heath when they are in unstable working conditions. Nonetheless, while one study argues that men are more unhealthy than women, most of the studies showed higher vulnerability in women as revealed in our study [12, 27, 31].

### Implication for the future

To tackle health inequities among young people belonging to a non-working status category was complicated. Indeed, as previously demonstrated by the WHO into the micro-conceptual framework and Benach et al. through their conceptual model putting precarious employment as a central issue, several factors should be considered simultaneously by policies, which makes these policies harder to be effective [3, 16].

On the other hand, to base strategies on the reinforcement of the protective effect of education appeared to be relevant. It was especially the case for alcohol consumption for which the protective effect seemed to be lower at university. Prevention policies should also take into consideration that young people in training school did not have the same protective effect as secondary school and university, for tobacco and marijuana consumption, in particular.

Individual life trajectories, including major event during childhood seemed to be a significant point to consider.

### Strength and limitation

The present work was a multicentre survey (115 LSC and 74 HEC) with a large sample (4282) of French young people. To our knowledge, this study was the sole which targeted young people in LSC. Occupational status was detailed in 13 categories but working conditions were not explored preventing us to assess deeply potential association between health outcomes and job characteristics such as number of hours worked per day, flexible employment, and if non-permanent job corresponds in personal choice or not. Individual employment trajectories, such as work linked to study field, type of work performed, were also not assessed accurately. Individual life trajectories were approached by the measurement of event during childhood. Furthermore, as young people who participated were volunteers, those in the worst life and health conditions could be not included.

## Conclusions

Occupational status of French young people was a determinant of health inequities. Young people in block release training school, training in education, undergoing integration into the work place, and job seeking, reported greater vulnerability to poor-self perceived health, addictions, poor mental health and violence. Young French people in training school or in block release training school do not benefit from occupational medicine service or university medicine service. This population should therefore be targeted by appropriate and specific social and medical services.

## Additional files

Additional file 1: Definitions about working according French rules. (PDF 66 kb)

Additional file 2: Questionnaire. (PDF 243 kb)

## Abbreviations

95%CI: 95% Confidence Interval
ADRS: Adolescent Depression Rating Scale
CETAF: Education Technical Centre of Health Examination Centres (Centre technique d’appui et de formation des centres d’examens de santé)
CNAMts: the French national health insurance for salaried workers (Caisse Nationale d’Assurance Maladie-travailleurs salariés)
CNML: National Council of Local Social Centres (Conseil National des Missions Locales-
DGAS: Social Welfare Directorate (Direction Générale de l’Action Sociale),
DGS: Health Directorate (Direction Générale de la Santé),
ESPAD: European School Survey Project on Alcohol and Other Drugs
HEC: Health Examination Centres
ILO: International Labour Organization
Insee: National Institute of Statistics and Economic studies
LSC: Local Social Centres
MHI-5: Mental Health Index-5 items
OFDT: French Monitoring Centre for Drugs and Drug Addiction (Observatoire Français des drogues et des Toxicomanies)
OR: Odds Ratio Support)
WHO: World Health Organization-Europe
