# Supplementary material for: Occupational status as a determinant of mental health inequities in French young people: is fairness needed? Results of a cross-sectional multicentre observational survey
Source: Int J Equity Health. 2017 Aug 8;16:142. doi: 10.1186/s12939-017-0634-7 (PMC5549318; doi:10.1186/s12939-017-0634-7)
Supplement: Supplementary file 1 — Definitions about working according French rules. (PDF 66 kb) [file 12939_2017_634_MOESM1_ESM.pdf]

Additional file 1 Definitions about working according French rules

English term	French term	Definition
Permanent job	Contrat à Durée Indéterminée (CDI)	
Temporary job	Contrat à Durée Déterminée (CDD)	
Interim	CDD dans le cadre d'une agence d'intérim	Temporary job managed by temping agencies
Specific employment contract	Contrat aidé	Temporary job supporting by the French State with reduced charge for the employer
School/secondary school	Collège/Lycée	
At university	Etudiants à l'université	
Out of university	Etudiants hors université	
Training school	En apprentissage	Specific education for apprentice in an apprentice training centre
Block release training school	Formation en alternance	School and work experience alternating
Trainee in education	Stagiaire de la formation professionnelle	Unemployment with registration in employment agency performing professional training
In integrating into the work place	En insertion	Unemployment with registration in employment agency and having specific contract for integration in social life
Job seeking	En recherche d'emploi	Unemployment with registration in employment agency and job seeking
Unemployed	Sans activité	Unemployment without registration in employment agency or struck off the employment agency
