# Supplementary material for: Occupational status as a determinant of mental health inequities in French young people: is fairness needed? Results of a cross-sectional multicentre observational survey
Source: Int J Equity Health. 2017 Aug 8;16:142. doi: 10.1186/s12939-017-0634-7 (PMC5549318; doi:10.1186/s12939-017-0634-7)
Supplement: Supplementary file 2 — Questionnaire. (PDF 243 kb) [file 12939_2017_634_MOESM2_ESM.pdf]

Additional file 2: Questionnaire

1. **Are you?** ☐ A man ☐ a woman
2. **How old are you?** ☐☐ years
3. **How many children do you have?** ☐☐ child(ren)
4. **Are you expecting a baby?** ☐ Yes ☐ No
5. **How many brothers and sisters do you have?** ☐☐
6. **What is the postal code of your home place?** ☐☐☐☐☐
7. **Where do you live the most often?**

   ☐ At your own home or in co-renting
   ☐ At your parent's home
   ☐ At relatives'home
   ☐ At friend's home
   ☐ At your boy/girl friend's home
   ☐ At your boy/girl friend parent's home
   ☐ Other, précised:
   ☐ At university housing
   ☐ At accommodation place (boarding school, hostel for young workers)
   ☐ At reception family's home
   ☐ Not at a home
   ☐ Homeless

8. **What is your current occupational status (several answers)?**

   ☐ School/Secondary school
   ☐ Specialized school
   ☐ Student at university
   ☐ Student out of university
   ☐ Training school
   ☐ Block release training school
   ☐ Trainee in education
   ☐ In integrating into the work place
   ☐ Job seeking
   ☐ Specific employment contract
   ☐ Temporary job
   ☐ Permanent job
   ☐ Interim
   ☐ No activity

9. What is your parents' current occupational status?

☐ Worker ☐ Invalid or long stop for disease

☐ Unemployed or job seeker ☐ Social allowance

☐ Training course ☐ Deceased

☐ Retired ☐ Do not know

☐ Housewife, off work after pregnancy

10. Do you have social follow up in local centre? ☐ Yes ☐ No

11. Are you the beneficiary of contract for social integration (Contrat d'insertion dans la vie sociale, CIVIS)? ☐ Yes ☐ No

12. Do you receive the solidarity income allocation (Revenu de solidarité active, RSA)? ☐ Yes ☐ No

13. Are you recognized as a handicapped person? ☐ Yes ☐ No

14. What is your current level of education?

☐Without diploma ☐Secondary school diploma + 2 years

☐ First year of training school ☐ University diploma

☐School diploma ☐ PHD

☐ Secondary school diploma

15. Do you have appointed a general practitioner as your gate keeper to the health insurance for salaried workers? ☐ Yes ☐ No ☐Do not know

16. Are you the beneficiary of universal health insurance (Couverture médicale universelle CMU)? ☐ Yes ☐ No ☐Do not know

17. Do you sometimes meet with a social worker (welfare worker, educator)? ☐ Yes ☐ No

18. Do you have complementary health insurance (mutual insurance)? ☐ Yes ☐ No

19. Do you live as a couple? ☐ Yes ☐ No

20. Are you a homeowner or will you be one in the near future? ☐ Yes ☐ No

21. Are there periods in the month when you have real financial difficulties in facing you need (food, rent, electricity, etc.)? ☐ Yes ☐ No

22. Have you participated in any sports activities in the last 12 months? ☐ Yes ☐ No

23. Have you gone to any shows (cinema, theatre) in the last 12 months? ☐ Yes ☐ No

24. Have you gone on holiday during the past 12 months? ☐ Yes ☐ No

25. Have you seen any family members in the past six months (other than your parents or children)? ☐ Yes ☐ No

26. Did you have difficulties (financial, family or health), is there anyone around you who could take you in for a few days? ☐ Yes ☐ No

27. Did you have difficulties (financial, family or health), is there anyone around you who could help you financially (material aid such as lending you money)? ☐ Yes ☐ No

28. During the last 30 days, do you have relation with yours parents? ☐ Yes ☐ No

29. Did you live these family events?

1. Break-up or divorce ☐ Yes ☐ No
2. Disease, injured, depression, alcoholism, serious accident of your father or mother ☐ Yes ☐ No
3. Break-up with your parents ☐ Yes ☐ No

30. Did you have an accident or a serious illness? ☐ Yes ☐ No

31. Did you be involved in an educative or judicial intervention? ☐ Yes ☐ No

32. What can tell about the childhood and adolescence you have lived (one answer)?

33. What is your current tobacco consumption?

34. Your alcohol consumption?

1. During the past 30 days, how many times did you drink alcohol?

None ☐ 1-2 times ☐ 3-5 times ☐ 6-9 times ☐ 10-19 times ☐ 20-29 ☐ ≥ 30 times ☐

2. During the past 12 months how many times did you were drunk?

None ☐ 1-2 times ☐ 3-5 times ☐ 6-9 times ☐ 10-19 times ☐ 20-29 ☐ ≥ 30 times ☐

35. During the past 30 days, did you use marijuana? ☐ Yes ☐ No

If yes, have you ever felt you needed to smoke marijuana to feel health since the morning? ☐ Yes ☐ No

36. Considering your age, please give a note between 0 and 10 to quote your self-perceived health

The worse health The best health

☐0 ☐1 ☐2 ☐3 ☐4 ☐5 ☐6 ☐7 ☐8 ☐9 10☐

37. What weight are you? ☐☐☐kg

38. How tall are you? ☐,☐☐m

39. These questions are about how you feel and how things have been with you during the past 4 weeks. For each question, please give the one answer that comes closest to the way you have been feeling.

How much of the time during the past 4 weeks...

1. Have you been very nervous?

☐ All of the time ☐Most of the time ☐ Some of the time ☐A little of the time ☐ None of the time

2. Have you felt so down in the dumps that nothing could cheer you up?

☐ All of the time ☐Most of the time ☐ Some of the time ☐A little of the time ☐ None of the time

3. Have you felt calm and peaceful?

☐ All of the time ☐Most of the time ☐ Some of the time ☐A little of the time ☐ None of the time

4. Have you felt downhearted and depressed?

☐ All of the time ☐Most of the time ☐ Some of the time ☐A little of the time ☐ None of the time

5. Have you been happy?

☐ All of the time ☐Most of the time ☐ Some of the time ☐A little of the time ☐ None of the time

40. You see above sentences collected from adolescents, please read each of them and stick “true” if it expresses something you are living or “false” if not.

1. I have no energy for work/school ☐ True ☐ False
2. I have trouble thinking ☐ True ☐ False
3. I feel overwhelmed by sadness and listlessness ☐ True ☐ False
4. Nothing really interests or entertains me ☐ True ☐ False
5. What I do is useless ☐ True ☐ False
6. When I feel this way I wish I were dead ☐ True ☐ False

7. Everything annoys me ☐ True ☐ False
8. I feel downhearted and discouraged ☐ True ☐ False
9. I sleep badly ☐ True ☐ False
10. School/work doesn't interest me just now, I can't cope ☐ True ☐ False

41. Have you ever thought about suicide even if it's not really seriously? ☐ Yes ☐ No

42. Have you ever thought about suicide seriously? ☐ Yes ☐ No

43. During the course of your life, did you commit suicide? ☐ Never ☐ once ☐ several time

If yes, did you go to hospital because of your suicide attempt? ☐Yes ☐No

44. During the course of your life, did you be victim of violence?

1. Psychological ☐Never ☐Once ☐Twice ☐3-4 times ☐5 times or more
2. Physical ☐Never ☐Once ☐Twice ☐3-4 times ☐5 times or more
3. Sexual ☐Never ☐Once ☐Twice ☐3-4 times ☐5 times or more

45. Have you ever be the perpetrator of a violent behaviour?

☐ None of the time ☐A little of the time ☐ Some of the time ☐Most of the time ☐ Very most of the time ☐ All of the time

46. Do you feel you are sometimes risked to violence? ☐Yes ☐No

If yes, in which place?

☐ At home ☐ At work ☐At school ☐In the street ☐ In public transport ☐In leisure centres

☐ Other: précised:

47. Do you think you are victim of discrimination to accede to

1. Employment? ☐Yes ☐No
2. Accommodation? ☐Yes ☐No
3. Leisure? ☐Yes ☐No
4. Education? ☐Yes ☐No
5. Transports? ☐Yes ☐No

If yes, it was the consequence of your:

☐ Origin, name ☐Sex

☐ Age

☐ Sexuality

☐Physical aspect

☐ Religion

☐ Health state, handicapped

☐ Location of your accommodation

☐ Other, précised:

48. During the past 12 months, because of nervous, psychological or of behaviour, did you met:

1. A general practitioner ☐Yes ☐No
2. A psychiatrist, psychologist, psychotherapist ☐Yes ☐No
3. A nurse ☐Yes ☐No
4. An other health care professional☐Yes ☐No
5. A social worker☐Yes ☐No
6. A friend or a member of your family ☐Yes ☐No
7. Other, ☐Yes ☐No

   précised:

49. How are you expected the future?

☐ Be hopeful and confident ☐Sometimes hopeful and sometimes worried ☐Very worried ☐Prefer to don't think about future

50. If you filled in this questionnaire in a health examination centre, did you be send to the health examination centre by the social centre? ☐Yes ☐No

51. Do you want to add something that is important for you?
